# GIS Analysis of Changes in Ecological Vulnerability Using a SPCA Model in the Loess Plateau of Northern Shaanxi, China

**DOI:** 10.3390/ijerph120404292

**Published:** 2015-04-17

**Authors:** Hou Kang, Li Xuxiang, Zhang Jing

**Affiliations:** School of Human Settlements and Civil Engineering, Xi’an Jiao tong University, Xi’an, 710049, China; E-Mails: houkang0311@stu.xjtu.edu.cn (H.K.); zhangjingzj0312@163.com (Z.J.)

**Keywords:** ecological vulnerability index, geographic information system (GIS), spatial principal component analysis, Northern Shaanxi area

## Abstract

Changes in ecological vulnerability were analyzed for Northern Shaanxi, China using a geographic information system (GIS). An evaluation model was developed using a spatial principal component analysis (SPCA) model containing land use, soil erosion, topography, climate, vegetation and social economy variables. Using this model, an ecological vulnerability index was computed for the research region. Using natural breaks classification (NBC), the evaluation results were divided into five types: potential, slight, light, medium and heavy. The results indicate that there is greater than average optimism about the conditions of the study region, and the ecological vulnerability index (EVI) of the southern eight counties is lower than that of the northern twelve counties. From 1997 to 2011, the ecological vulnerability index gradually decreased, which means that environmental security was gradually enhanced, although there are still some places that have gradually deteriorated over the past 15 years. In the study area, government and economic factors and precipitation are the main reasons for the changes in ecological vulnerability.

## 1. Introduction

The Northern Shaanxi agropastoral areas in the hilly-gully region of the Loess Plateau are typical of ecologically fragile areas where water and wind erosion are very serious problems. In recent years, energy exploitation and utilization have seriously affected the ecological balance of the area and made it sensitive to change. The ecological environment in Northern Shaanxi is more sensitive to stress or interference, and its geographical location means that its environment is unique on the Loess Plateau. A comprehensive eco-environment evaluation of the Northern Shaanxi region is urgently needed to improve ecological and environmental protection and governance because it would not only reveal the forces driving eco-environmental change but also shape land use policies to restore and expand ecosystem services and mitigate the ecological effects of radiation [1]. In other words, a comprehensive eco-environment evaluation would be the basis for taking effective measures to control the deterioration of the ecological environment and effectively prevent the unreasonable destruction of the socio-ecological environment due to the effects of human activities as well as protect China's energy and chemical resources and ensure the safety of the Yellow River downstream. It also provides the theoretical and scientific basis for regional eco-environmental development in ecologically fragile areas [2,3].

As an important aspect of environmental assessment, the concept of ecological vulnerability has been developed in recent years. The concept of vulnerability is derived from the social sciences, but there is no general agreement over how to define vulnerability as part of an environmental impact assessment [4]. Research into ecological vulnerability, which originated from a basic definition by the IPCC, has become an important aspect of research on global environmental change and sustainable development [5]. Although the assessment of ecological vulnerability is a relatively new field that has developed rapidly in recent years, ecological restoration continues to be a source of useful information [6,7]. A variety of evaluation methods has been developed, such as the Integrated Assessment Act (IAA) [8], the analytical hierarchy process (AHP) [9] and the forecast weighting method (IWM) [10]. However, these methods have been established for experts to assess the importance of the factors under consideration that will directly lead to the results of the final evaluation [4,10,11]. This paper selected spatial principal component analysis (SPCA) to evaluate the ecological vulnerability of the Northern Shaanxi region.

Recently, remote sensing (RS) and geographic information systems (GIS) have emerged as powerful tools to support ecological vulnerability assessments [12]. Satellite images are especially valuable because they provide frequently updated maps of inaccessible areas or areas with rapidly changing landforms [13,14,15]. The integration of RS with GIS provides an excellent framework for data capture, storage, synthesis, measurement, and analysis, all of which are essential to eco-environmental analysis [1]. An evaluation system includes natural environmental factors, social factors and human factors, and in this study, GIS was used in the eco-environmental evaluation model to provide an objective assessment of ecological vulnerability [16,17,18]. The objectives of this study were: (1) to develop a relatively reasonable evaluation system using GIS; (2) to establish a regional ecological vulnerability assessment model based on spatial principal component analysis (SPCA); (3) to analyze changes in ecological vulnerability since the implementation of the policy to return farmland to forest, and (4) to analyze and improve our understanding of the factors driving eco-environmental changes so that a sustainable land use strategy can be established.

## 2. Study Area and Data

### 2.1. Study Area

**Figure 1:**
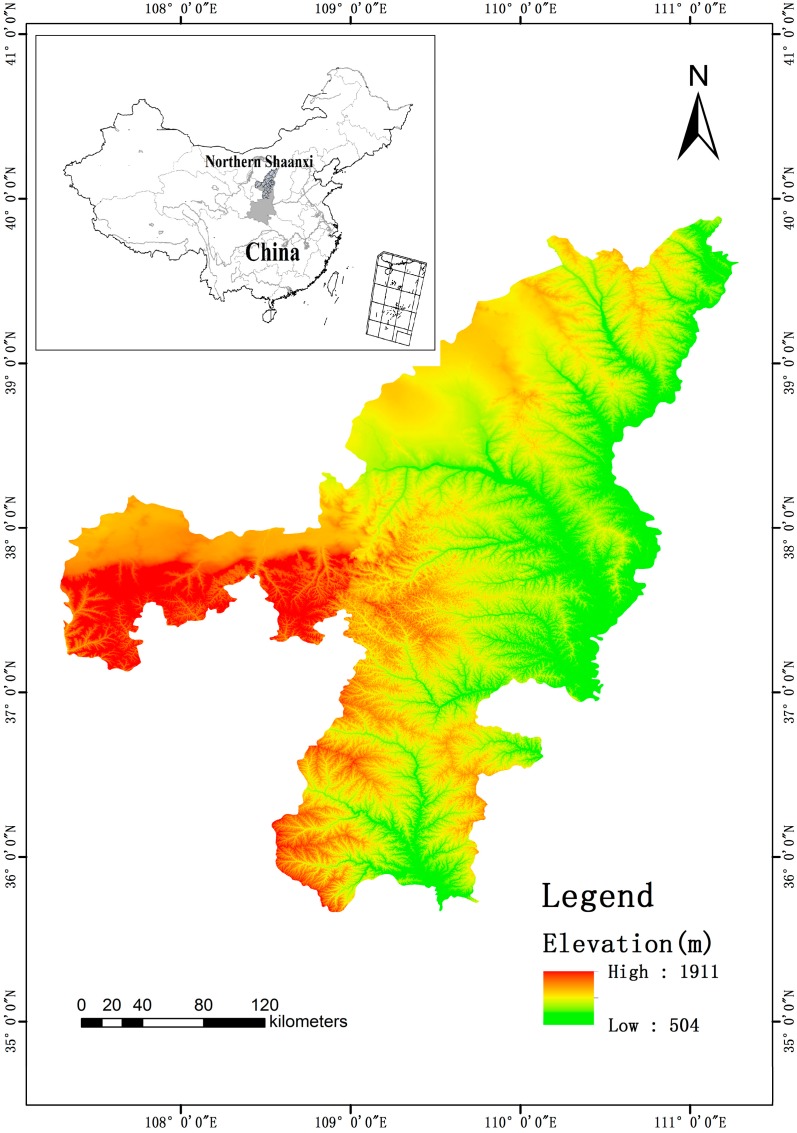
The location of the study area.

The Northern Shaanxi region was selected as the study area to conduct this research. It is located in the middle reaches of the Yellow River, which marks the border between the Loess Plateau and the southern zone of the Mu Us Desert (see Figure 1). The study area is confined by 35°34′~39°58′ N latitude and 107°33′~111°24′ E longitude. According to the administrative boundaries, the study area involved two regions, namely, the Yulin Region (including Yuyang District, Fugu County, Shenmu County, Dingbian County, Jingbian County, Hengshan County, Mizhi County, Jiaxian County, Zizhou County, Wubu County, Suide County and Qingjian County) and the Yan’an Region (including Baota District, Ansai County, Zichang County, Yanchuan County, Ganquan County, Fuxian County, Luochuan County, and Huangling County).

### 2.2. Data and Selection

The core data consisted of Landsat-5 TM remote sensing images from a specific time in June of 1997, 2004 and 2011, which have a spatial resolution of 30 meters, and serial numbers 127, 33; 127, 34 and 128, 34, respectively. The remote sensing data were obtained from the United States Geological Survey (USGS), and the map projection coordinates were in Universal Transverse Mercator (UTM). Meanwhile, in addition to the remote sensing data, we also acquired natural, economic and social data from the 1997, 2004 and 2011 Statistical Yearbooks and relevant government departments.

Land use type, altitude, vegetation cover and forest area were interpreted from Landsat resource thematic mapper (TM) images, and industrial output, agricultural output, population density, soil erosion area and *per capita* GDP were obtained from the Statistical Yearbook of Shaanxi Province of the corresponding year. Moreover, rainfall, hours of sunshine and average annual temperature were obtained from the Shaanxi Meteorological Bureau of the Agricultural Remote Sensing Center.

The selection of evaluation criteria plays a key role in a regional ecological assessment. The evaluation factors should be operational, indicative, and representative [19,20]. Various factors influencing the ecological vulnerability of the Loess Plateau are considered. Based on some previous qualitative analyses of ecological features in the study area, we considered all possible environmental variables for the present assessment. All variables were submitted to principal component analysis (PCA) to reduce data dimensionality by performing a covariance analysis between factors. The 12 independent variables representing the principal trait of the environmental variability are selected to assess the ecological vulnerability for the study area. Natural conditions including topography, altitude, climate, and vegetation cover form an important determinant of vulnerability evaluation [21]. Soil erosion, land use and forest are considered because the study area is severely suffering from these earth surface processes and environmental problems [22]. The regional environmental vulnerability is also strongly related to local socio-economic factors since human activities can greatly influence the evolution of numerous environmental characteristics [23]. Population density, *per capita* GDP and cultivation and exploitation disturbances are therefore selected to evaluate the impacts of human activities. Generally, cultivation and exploitation disturbances can be reflect by agricultural and output and industrial output. In this region, the impact of industrial or agricultural plays a major role. 

## 3. Methods

### 3.1. Evaluation Factors and Data Standardization

In the ecological vulnerability assessment system, the choice of evaluation criteria is crucial; criteria should be representative and adaptable. Meanwhile, the ecological environment is a dynamic, balanced system that is constantly affected by changing energy and material cycles. Therefore, the factors used for assessment in the quantitative study should consider natural environmental variables and the impacts of human activity. The evaluation system in this study included 12 variables: land use, rainfall, annual sunshine time, average annual temperature, population density, per capita GDP, altitude, vegetation cover, forest area, industrial output, agricultural output, and soil erosion area.

Because of the different units used for the values of the assessment factors, it is difficult to directly evaluate the status of the eco-environment as these values must be standardized to reflect a uniform measurement system across all factors. The original values of each variable were standardized using SPSS18.0 software by the following equation:

(1) $$Y_{ij}=\frac{x_{ij}-x_{\min,j}}{x_{\max,j}-x_{\min,j}}\times 10$$

where *Y_ij_* represents the standardized value of factor *j* of unit *i* and varies from 0 to 10; *x_ij_* represents the measured value of factor *j* of unit *i*, and x_min,*j*_ and x_max,*j*_ represent the minimum and maximum values of factor *j* of unit *i*.

### 3.2. Evaluation Model

Converting the factors into an integrated evaluation index is a key step in environmental evaluation and remains difficult. Generally, the observed data for each variable presents a problem in that certain variables will correlate, *i.e.*, the observation data and the information they reflect will have a certain amount of overlap [24,25]. Through different dimensions of data standardization, principal component analysis (PCA) can compress the data set and transform the index data into a variety of representative, comprehensive data [21,26]. The characteristics of each variable that reflect this comprehensive feature can then be analyzed. The principal components provide information about the most meaningful parameters, which are those that describe the whole data set and allow for data reduction with minimal loss of the original information. PCA can be expressed as follows:

(2) $$Y=n_{1}x_{1}+n_{2}x_{2}+...+n_{m}x_{m}$$

where *Y* is the component score; *n* is the component loading; *x* is the measured value of a variable; *i* is the component number, and *m* is the total number of variables.

PCA can therefore be used as descriptive, statistical approach to data transformation as a means of overcoming variable incommensurability. The ranking of the PCs in order of their significance (based on how much of the variability in the data they capture) is denoted by the eigenvalues associated with the vector for each PC. In the case of a spatially explicit analysis each data point for each variable is related to a specific point in space and the PCs derived from a PCA can be assigned scores (synthetic variable values) for each of these points in space. Demšar [26] had a very detailed analysis on operational processes and applications of the PCA. In the “Raster Data PCA” section, it is actually the SPCA method by using ArcGiS software. Therefore, SPCA also is a modified PCA method.

This study used an ecological environmental vulnerability evaluation model based on the SPCA method. Spatial principal component analysis (SPCA) transforms the attributes in multiband spatial data into a new multivariate attribute space whose axes are rotated with respect to the original space so that the axes in the new space are uncorrelated. The result of SPCA is a new multilane spatial data set with the same number of bands as the original data. SPCA has certain advantages over conventional orthogonal functions because the resulting data are not of any predetermined form but are developed as unique functions from the data matrix. This is particularly useful if nothing is known in advance about the existence or nature of the patterns of the components [27], so SPCA was used to evaluate ecological vulnerability in this study.

The higher the EVI value, the more vulnerable the ecological environment is. The EVI is defined as the sum of a couple of weighted principal components as shown below:

(3) $$EVI=n_{1}F_{1}+n_{2}F_{2}+...+n_{m}F_{m}$$

where *EVI* is the eco-environmental synthetic evaluation index; *n* is the contribution ratio of the principal component; *F* is the principal component, and m is the number of principal components retained:

(4) $$n_{i}=\frac{r_{i}}{\sum_{i=1}^{m}r_{i}}$$

where *n*_i_ is the contribution ratio of the *i*th principal component, and *r*_i_ is the eigenvalue of the *i*th principal component.

### 3.3. Vulnerability Gradation

The EVI was computed from the SPCA assessment model, and it was then classified into several ranks to represent the different levels of ecological vulnerability. The natural breaks classification (NBC) is an objective and rational measure to explore the statistical distribution of classes and clusters in an attribute space using ArcGIS 9.3 software [28]. The NBC can identify the break points that group similar values and maximize the differences between classes, and the features are then divided into classes whose boundaries are the relatively large gaps between values. In this study, the NBC was used to divide the ecological vulnerability assessment into five levels: potential, slight, light, medium, and heavy.

## 4. Results

### 4.1. Eco-Environmental Vulnerability Assessment of 1997, 2004 and 2011

According to the correlation analysis, all 12 of the factors selected for the ecological vulnerability assessment represent important aspects of the environment, and the SPCA identified four principal components. The cumulative contribution results of the principal components are shown in Table 1.

**Table 1:** The results of spatial principal component analysis

	Principal Component	1997a	2004a	2011a
Eigenvalue(n)	I	4.032	3.109	3.583
	II	2.657	2.571	2.392
	III	2.002	2.087	1.363
	IV	1.129	1.042	1.075
Contribution (%)	I	33.602	28.261	32.569
	II	22.144	23.375	22.749
	III	16.687	18.973	14.392
	IV	9.412	9.469	9.775
Cumulative contribution (%)	I	33.602	28.261	32.569
	II	55.746	51.636	55.318
	III	72.433	70.608	69.709
	IV	81.845	80.077	79.485

As derived from Table 1 and Equations (3) and (4), the ecological vulnerability assessment index (EVI) of the northern Shaanxi region is defined as the sum of a couple of weighted principal components as follows:

(5) $$EVI_{1997}=0.4106A_{1}+0.2706A_{2}+0.2039A_{3}+0.1150A_{4}$$

(6) $$EVI_{2004}=0.3529B_{1}+0.2919B_{2}+0.2369B_{3}+0.1183B_{4}$$

(7) $$EVI_{2011}=0.4259C_{1}+0.2843C_{2}+0.1620C_{3}+0.1278C_{4}$$

In the equations, the EVI is the synthetic ecological vulnerability evaluation index, and *A*1~*A*4 are the four principal components from the twelve initial spatial variables in 1997. Likewise, *B*1~*B*4 are the four principal components in 2004, and *C*1~*C*4 are the four principal components in 2011.

**Figure 2:**
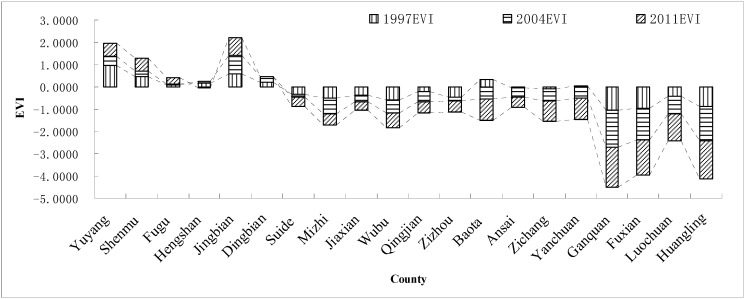
The ecological vulnerability of Northern Shaanxi in 1997, 2004 and 2011.

Using Equations (5‒7), the synthetic ecological vulnerability index of the study area was calculated (see Figure 2). In 1997, the EVI of Yuyang District, Jingbian County and Baota District was larger than the EVI of the other counties, and in 2004, the EVI of Yuyang District and Jingbian County was still larger than the EVI in the other counties. In 2011, the trend was similar to 2004. From 1997 to 2011, the EVI of Ganquan County, Fuxian County and Huangling County was smaller than the EVI in the other counties. Overall, the EVI in the Yulin Region is larger than the Yan’an Region.

### 4.2. Distribution of the Ecological Vulnerability Grades

**Figure 3:**
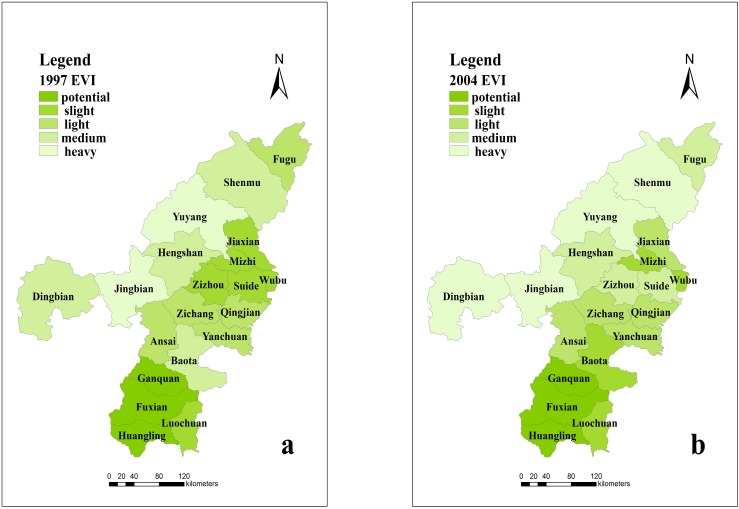

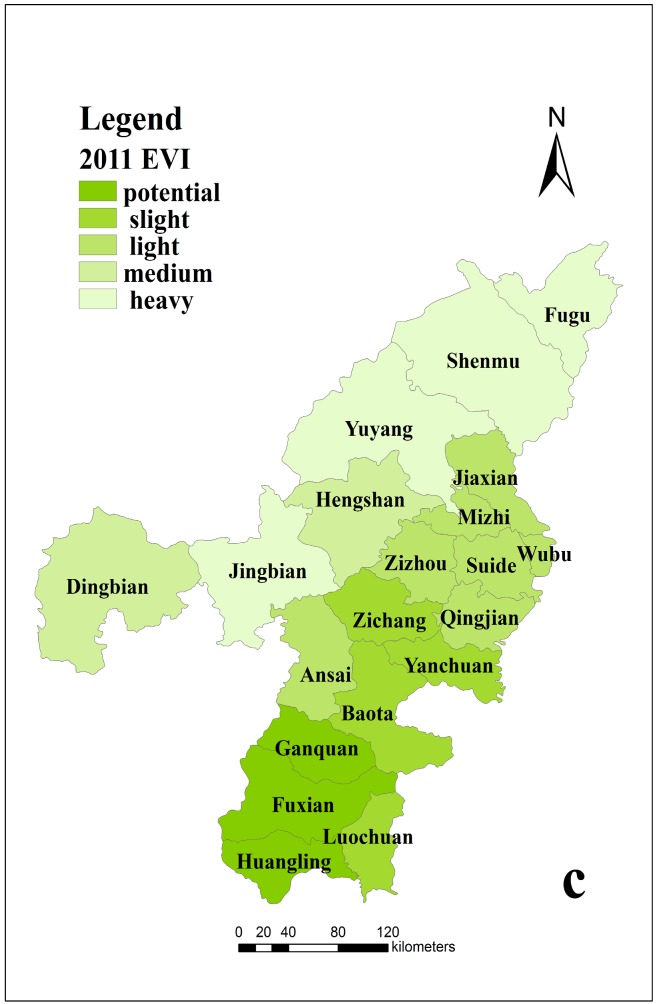
The ecological vulnerability of Northern Shaanxi in 1997 (**a**), 2004 (**b**) and 2011 (**c**).

The natural breaks classification method divided the ecological vulnerability data from the study area into five grades: potential, slight, light, medium and heavy (see Table 2). The results of the assessment and grading can be used to reflect the actual local eco-environmental conditions in Northern Shaanxi (see Figure 3). The ecological vulnerability in most of the counties was at the level of potential, slight or light, but some counties were at medium or heavy levels. 

In 2000, the heavily vulnerable zones were mainly distributed in the Yuyang District, Jingbian County, and Baota District, and the potentially vulnerable areas were mainly distributed in Fuxian County and Ganquan County. In 2004, the potentially vulnerable areas were mainly distributed in Ganquan County, Fuxian County and Huangling County, and the heavily vulnerable areas were mainly distributed in the Yulin Region. In 2011, the distributions of the potential and heavy areas were consistent with their distributions in 2004.

**Table 2:** Classification of ecological vulnerability in Northern Shaanxi

Evaluation Level		EVI	Feature Description	
Potential		−1.8000~−0.8800	Stable ecosystem, extremely high anti-interference ability, rich soil, abundant water and heat, and good vegetation cover	
Slight		−0.8799~−0.3394	Relatively stable ecosystem, high anti-interference ability, rich soil, abundant water and heat, and relatively good vegetation cover	
Light		−0.3393~0.0947	Relatively stable ecosystem, relatively high anti-interference ability, infertile soil, and relatively poor vegetation cover	
Medium		0.0948~0.4507	Relatively unstable ecosystem, low anti-interference ability, poor-quality soil, and poor vegetation cover	
Heavy		0.4508~0.9546	Unstable ecosystem, low anti-interference ability, deteriorated soil, and poor vegetation cover	

## 5. Discussion

### 5.1. Analysis of the Changes in Ecological Vulnerability

**Figure 4:**
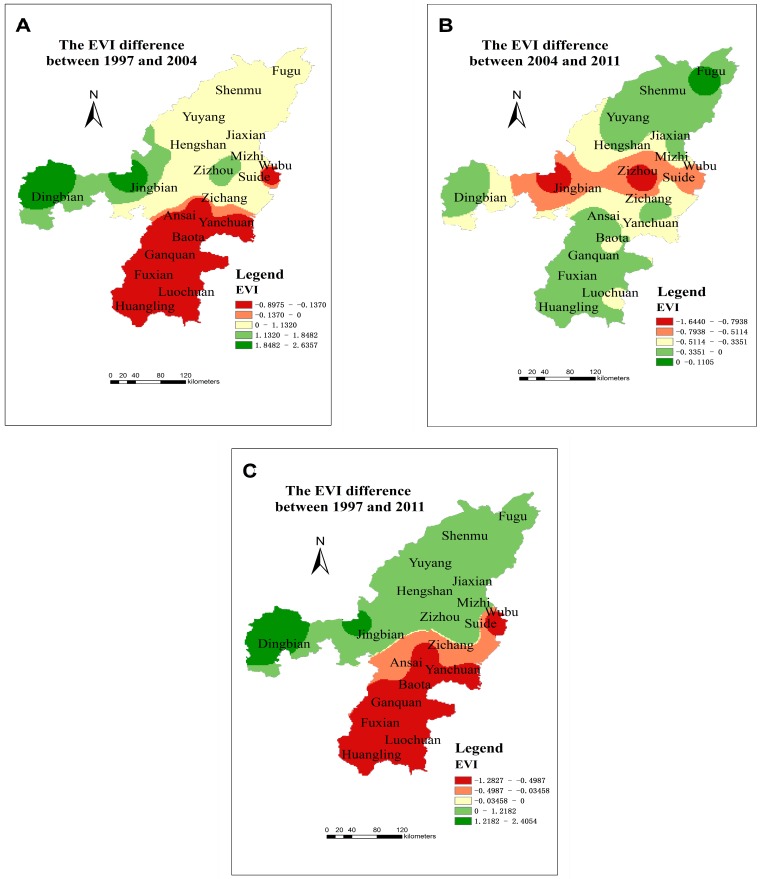
The changes in EVI difference between 1997 and 2004 (**A**); between 2004 and 2011 (**B**); between 1997 and 2011 (**C**).

From 1997 to 2011, the overall ecological vulnerability index decreased in Northern Shaanxi, which means that the ecological environment was gradually stabilizing, but the ecological vulnerability index gradually increased in some counties, specifically in the Yulin Region (see Figure 4). However, Hengshan County, Dingbian County and Qingjian County were abnormal because the EVI in later years was larger. The EVI of Baota District, Ansai County, Zichang County, Yanchuan County, Ganquan County, Fuxian County, Luochuan County and Huangling County gradually decreased from 1997 to 2011, so ecological security gradually increased. From 1997 to 2004, the ecological vulnerability index decreased in southern area, but most northern areas of ecological vulnerability index has gradually increased. From 2004 to 2011, the ecological vulnerability index decreased in the central region of the study area, and other areas of ecological vulnerability index had a small increase.

Spatially, the range of the ecological vulnerability index values of the study area gradually decreased from north to south (see Figure 3 and Figure 4). The EVI of the twelve northern counties (including Yuyang District, Fugu County, Shenmu County, Hengshan County, Jingbian County, Dingbian County, Suide County, Mizhi County, Jiaxia County, Qingjian County, Wubu County and Zizhou County) was significantly higher than that for the eight southern counties (including Baota District, Ansai County, Zichang County, Yanchuan County, Ganquan County, Fuxian County, Luochuan County and Huangling County). In other words, ecological security gradually became stronger from north to south.

The ecological vulnerability index of Ganquan County and Huangling County was the lowest, which illustrates the ecological security and stability of the region. In the research region, the ecological vulnerability index of Yuyang District, Shenmu County, and Jingbian County was higher than that of the other counties, and the exploitation of oil and coal was one of the important factors.

### 5.2. Evaluation of the Model and the Results

The use of GIS technology can provide more accurate results when assessing environmental vulnerability at regional or national scales as well as easily create graphics to display the spatial distribution of complex environmental characteristics. Moreover, the contribution of this study is that we added some of the socio-economic factors that influence environmental vulnerability to the model. Our case study demonstrated that the proposed method is effective, and it can also be used to assess the environmental vulnerability of other regions. The study area is typical of the hilly-gully region in the Loess Plateau, and the general ecological vulnerability grade of the study region is light, which is consistent with local ecological conditions. Meanwhile, the EVI in Northern Shaanxi varies geographically; it gradually decreases from north to south, which demonstrates that the evaluation results represent regional features and status.

### 5.3. The Analysis of the Driving Factors

In the Northern Shaanxi area, the ecological vulnerability index gradually decreased over time, which reflects that eco-environmental quality improved throughout most of the area. However the ecological vulnerability index of some counties increased from 1997 to 2000, and there are many factors that can affect eco-environmental stability. In general, energy development and the policy of returning farmland to forest and natural vegetation were the main forces driving the changes in ecological vulnerability.

Northern Shaanxi is China’s national energy and chemical base, so economic development mainly depends on energy development. Energy exploitation directly causes soil erosion in ecologically fragile areas, and it can directly affect the environment [29]. In recent years, the Chinese government has paid more attention to the control of energy exploitation and sustainable development in the Loess Plateau of Northern Shaanxi. More slopes have been converted from fields to grassland and woodland in past decade [30], and in 1999, the Chinese government began to implement a policy of returning farmland to forest, which is one of the largest conservation projects in the word. The policy requires farmland with slopes greater than 25° to be converted to woodland and grassland and as more and more slopes have been converted, the quality of the environment has improved. Therefore, in recent years, the ecological vulnerability index has decreased in most of Northern Shaanxi.

Vegetation is an important factor affecting the ecological balance, especially in ecologically fragile areas. Many areas in Northern Shaanxi have serious soil and water erosion problems, and the vegetation has been degraded. The mining of coal and oil also seriously damages the vegetation. Due to the limitations of natural ecological recovery, the effect of returning farmland to forests is not obvious in some places, such as in Shenmu County, Fugu County and Jingbian County. Therefore, developing a reasonable vegetation restoration program is the focus of future work as it directly affects the degree of ecological vulnerability.

## 6. Conclusions

Using Northern Shaanxi as a case study, this paper analyzed long-term (from 1997 to 2011) ecological vulnerability. Moreover, by incorporating a measure of ecological stability, changes in ecological vulnerability were analyzed following the implementation of a policy to return farmland to forest.

By comparing the ecological vulnerability in 2004 and 2011 with that of 1997, the potentially vulnerable, slightly vulnerable and lightly vulnerable areas all increased from 1997 to 2011, and these results reflect a gradual improvement in eco-environmental quality in most places. However, there are still some places that have gradually deteriorated over the past 15 years. There are many factors that affect ecological vulnerability, but energy development, policy and vegetation were the main driving forces affecting ecological vulnerability.

Finally, this study considered multiple factors in its assessment of regional environmental vulnerability and used an SPCA model to closely reflect the real situation of the study area. Compared to traditional estimation methods, this approach allows us to integrate various types of spatial information and socio-economic data. However, due to limited data and technical problems, this study did not evaluate the environment in specific energy exploitation areas. Therefore, more attention should be paid to this region in future research to provide reasonable suggestions for ecological recovery and sustainable development in this ecologically vulnerable region.
